# Nutritional Strategies Facing an Older Demographic: The Nutrition UP 65 Study Protocol

**DOI:** 10.2196/resprot.6037

**Published:** 2016-09-14

**Authors:** Teresa F Amaral, Alejandro Santos, Rita S Guerra, Ana S Sousa, Luísa Álvares, Rui Valdiviesso, Cláudia Afonso, Patrícia Padrão, Cátia Martins, Graça Ferro, Pedro Moreira, Nuno Borges

**Affiliations:** ^1^ Faculdade de Ciências da Nutrição e Alimentação da Universidade do Porto Porto Portugal; ^2^ Departamento para a Pesquisa do Cancro e Medicina Molecular, Universidade Norueguesa de Ciência e Tecnologia Trondheim Norway; ^3^ Unidade Local de Saúde do Alto Minho, EPE Viana do Castelo Portugal

**Keywords:** older adults, nutritional inequalities, Portugal

## Abstract

**Background:**

The population of Portugal is aging. The lack of data on older adults’ nutritional status and the lack of nutrition knowledge amongst health professionals, caregivers, and older adults themselves, remains a challenge.

**Objective:**

The Nutrition UP 65 study aims to reduce nutritional inequalities in the older Portuguese adult population and improve knowledge regarding older Portuguese adults’ nutritional status, specifically relating to undernutrition, obesity, sarcopenia, frailty, hydration, sodium, and vitamin D statuses.

**Methods:**

A representative sample of older Portuguese adults was selected. Sociodemographic, lifestyle, anthropometric, functional, and clinical data were collected. Sodium excretion, hydration, and vitamin D statuses were assessed.

**Results:**

Data collection (n=1500) took place between December, 2015 and June, 2016. Results will be disseminated in national and international scientific journals, and via Portuguese media.

**Conclusions:**

Nutrition UP 65 results will provide evidence for the design and implementation of effective preventive public health strategies regarding the elderly. These insights may represent relevant health gains and costs savings.

## Introduction

Demographic projections for the 28 member states of the European Union show that as the population continues to age, the population aged >65 years is estimated to increase from 17% in 2008 to over 25% in 2035, and to 30% in 2060 [1]. As this older population ages, it is expected that the proportion of people aged >80 years will increase from 4.4% in 2008 to 12.1% in 2060 [1]. Accordingly, the Portuguese population is also getting older. Data from the most recent national census in 2011 revealed that 19% of the population was >65 years, and there was an increase of 18.7% in the older population between 2001 and 2011 [2].

These projections are of major concern, due to the links between aging and cognitive and functional decline, emotional changes, and depressive symptoms (all of which may directly influence general health) and, in particular, nutritional status. Furthermore, the scarce national data that is available in Portugal reveals that older adults’ nutritional inequalities are present in an accentuated way [3]. The majority of older Portuguese adults have economic constraints, which directly impact on food security [3]. The current socioeconomic situation in countries experiencing an economic crisis (such as Portugal) leads us to predict that the frequency and consequences of nutritional status-related disabilities will increase in the coming years.

Frail elders living in the community, institutionalized in nursing homes, or admitted to hospitals have increased risks associated with nutritional disorders [4]. In many instances the existing nutritional disorders of these patients go unrecognized and adversely affect their clinical outcomes. Nutritional status impairment in older adults is a serious public health problem [5,6]. Despite the alarming data released during the last decade relating to the negative influence of nutritional disorders on the health status of older populations, undernutrition occurrence is still very common in Europe [7,8]. Data from a systematic sample of patients admitted to six Portuguese hospitals showed that undernutrition is prevalent, affecting approximately one in three patients upon admission [9]. Undernutrition is a relevant factor for disease prognosis and is linked to higher odds of morbidity, premature mortality, and higher costs of care [10]. In addition, older age is an established risk factor for undernutrition [9]. Data regarding the prevalence of undernutrition and general nutritional status of older Portuguese adults living in communities are scarce and limited to a small number of geographic areas in Portugal [11]. Additional knowledge about the dimensions of undernutrition frequency in different regions, as well as the identification of the main factors associated with this problem, will allow for a better design and implementation of preventive strategies.

In addition to undernutrition, other priority areas will be addressed in this project. Nutritional status of fat-soluble vitamins in subjects aged >65 years is highly variable and determined by season, nutritional status, inflammation, renal function, and hospitalization [12]. The skin of elderly people produces less vitamin D than the skin of younger people; moreover, older adults also spend less time in the sun, and this population has an increased risk of vitamin D deficiency [13]. Data from a European report revealed a prevalence of vitamin D deficiency of up to 40% [14], and vitamin D deficiency among institutionalized and/or hip fracture patients is a major concern [15]. However, there is a lack of knowledge regarding the burden of vitamin D deficiency in Portugal.

Dehydration is a common condition among older people, and likely contributes to a number of medical conditions that lead to higher morbidity and mortality in these individuals [16]. Despite the scarcity of data pertaining to the hydration status of the Portuguese population, the assessment of fluid intake in a representative sample of Portuguese adults revealed a low intake of fluids by older subjects, particularly elderly men that reported to have consumed 51% less fluids than the recommended intake [17].

The World Health Organization recommends no more than 2 grams of sodium (5 grams of salt) per day for adults, in order to reduce the burden of noncommunicable diseases [18]; however, in all countries with recent data available, salt intake is much higher than recommended [19]. Excessive sodium intake is strongly associated with high blood pressure [20] and approximately 75% of the Portuguese elderly have been classified as hypertensive [21]. To our knowledge, the estimated amount of sodium ingested by this Portuguese subpopulation has not been published.

Current trends also indicate that the prevalence of obesity and sarcopenic obesity in this age group is increasing [22,23]. These conditions also have implications for the frailty of the elderly, which is strongly associated with higher mortality in older adults [24]. Nevertheless, Portuguese data concerning the dimensions of these conditions is scarce.

Together with the aforementioned trends and data, the absence of adequate nutritional data in Portugal (particularly in settings such as community and care institutions) reinforces the relevance of this study. The main objective of the Nutrition UP 65 study is to expand the knowledge of older Portuguese adults’ nutritional status. More specifically, the study aims to improve the information regarding undernutrition, obesity, sarcopenia, frailty, hydration, sodium and vitamin D statuses. These data will be a basis for the development of public health guidelines, with the goal of reducing nutritional inequalities in the older Portuguese population.

## Methods

### Study Design and Setting

A cross-sectional observational study was conducted in Portugal in a cluster sample of 1500 older adults (≥65 years old), which was representative of the older Portuguese population in terms of age, sex, education, and regional area. Data from the most recent national census in 2011 showed that the number of Portuguese residents was 10,562,178 and a total of 2,010,064 older Portuguese adults were identified, corresponding to 19% of the Portuguese population [2]. Thus, the recruited study sample (n=1500) corresponds to 0.075% of the Portuguese older population. Data for this study were collected between December, 2015 and June, 2016.

### Ethics

This research was conducted according to the guidelines established by the Declaration of Helsinki, and the study protocol was approved by the Ethics Committee of the department of Ciências Sociais e Saúde (Social Sciences and Health) from the Faculdade de Medicina da Universidade do Porto (PCEDCSS – FMUP 15/2015) and by the Portuguese National Commission of Data Protection (9427/2015).

### Sampling and Recruitment

To achieve a nationally representative sample of older Portuguese adults aged ≥65 years, a quota sampling approach was adopted using data from Census 2011 regarding sex, age, educational level, and residence area.

Individuals were considered to be Portuguese if they had only Portuguese nationality and if their current tax residence was in Portugal, and were eligible to participate in the study if they were aged ≥65 years. The following age categories were considered: 65-69, 70-74, 75-79, 80-84, 85-89, and >90 years old [25]. Educational level was determined by the number of school years completed, and the following categories were used: <4 years of schooling, first cycle (4 years of schooling), second cycle (6 years of schooling), third cycle (9 years of schooling), secondary (12 years of schooling), post-secondary (>12 years of schooling but no higher education), and higher education (academic, vocational, and advanced professional education) [25]. The regional areas used were defined in the Nomenclature of Territorial Units for Statistics: Alentejo, Algarve, Azores, Lisbon Metropolitan Area, Center, Madeira, and North (Multimedia Appendix 1) [26].

A random, stratified, and clustered sampling method was applied. In each regional area, three or more town councils with >250 inhabitants were randomly selected. Potential community-dwelling participants were contacted via home approach, telephone, or via institutions, such as town councils and parish centers.

**Figure 1:**
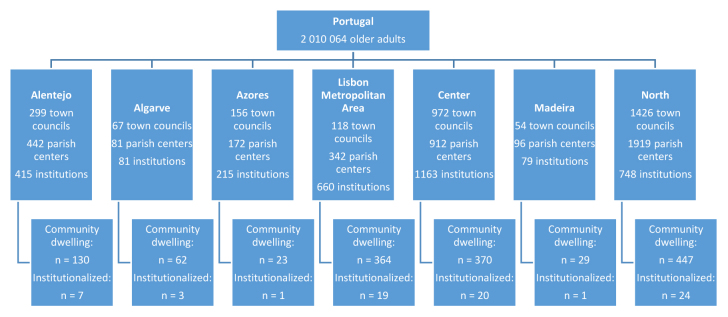
Sample composition and distribution.

The study sample was composed of community-dwelling older adults and individuals institutionalized in retirement homes, representing the 5% proportionality of older Portuguese individuals [2]. Participants were considered to be community-dwelling individuals if she/he slept in their own house, or in the house of a family member or friend, more than half of the days of the preceding month. Individuals institutionalized in retirement homes were contacted through the individual institutions (Figure 1).

Potential participants were contacted by the interviewer, who provided information about the study purposes and methodology, and invited them to participate. A document entitled *Information for the participant* was prepared and read by each potential participant or by a surrogate. In cases of acceptance, all participants (or two representatives if the participant was deemed to be cognitively impaired) were asked to read and sign a duplicated *Informed consent* form. Individuals presenting any condition that precluded the collection of venous blood samples or urine (eg, dementia or urinary incontinence) were excluded from the study.

### Sociodemographic, Anthropometric, Lifestyle, and Clinical Data

Demographic data, cognitive performance, current and former professional occupation, lifestyle practices, health status and clinical history, nutritional status, cohabitation, skin phenotype, and household income were collected using a structured questionnaire applied by means of an interview. The interview was conducted by eight previously trained registered nutritionists, who were also responsible for anthropometric data collection.

Demographic data included sex, date of birth, marital status, and education. Cognitive performance was assessed by the Portuguese version of the Mini Mental State Examination [27]. This test consists of 30 questions (each scored one point if correct) and examines the functions of orientation, registration, attention and calculation, recall, language, and ability to follow simple commands. The cutoff scores for cognitive impairment are as follows: individuals with no education, ≦15 points; 1 to 11 years of years of school completed, ≦22 points; and ≦11 years of school completed, ≦27 points. For individuals identified as presenting cognitive impairment, the *Informed consent* form was signed by two representatives and all data was provided by a person close to the participant, such as a family member or caregiver.

Lifestyle was evaluated via involvement in physical activities during the past seven days, current and former tobacco use, consumption of alcoholic beverages, and adherence to the Mediterranean diet, as described below.

Physical activity was assessed by the short form of the International Physical Activity Questionnaire [28]. This questionnaire gathers information regarding the previous seven days, namely how many days and how much time the participant spent: walking or hiking (at home or at work, moving from place to place, for recreation or sport), sitting (at a desk, visiting friends, reading, studying, or watching television), moderate activities (carrying light objects, hunting, carpentry, gardening, cycling at a normal pace, or tennis with two pairs), and vigorous activities (lifting heavy objects, agriculture, digging, aerobics, swimming, playing football, or cycling at a fast pace).

Adherence to the Mediterranean diet was evaluated with the Portuguese version of the Prevention with Mediterranean Diet tool [29]. This tool was developed with the purpose of testing the effectiveness of the Mediterranean diet on the primary prevention of cardiovascular disease, and consists of 14 questions, each scored with zero or one point. The criteria for assigning one point are established and a final score ≥10 indicates a good adherence to the Mediterranean diet.

Data regarding subjective general health were collected using questions drawn from the Portuguese National Health Survey 2005-2006. These questions concerned: self-reported diagnosis of chronic diseases in the past 12 months, namely the presence of asthma; chronic bronchitis, chronic obstructive pulmonary disease, or emphysema; myocardial infarction or chronic consequences of myocardial infarction; coronary heart disease or angina pectoris; hypertension; stroke or chronic consequences of a stroke; arthrosis; lumbar pain or other chronic lumbar problems; neck pain or other chronic neck problems; diabetes; hepatic cirrhosis; allergies; chronic renal disease, including renal failure; urinary incontinence or bladder control problems; depression and other diseases; and pharmacological treatment and use of nutritional supplements, including the name and number of daily doses.

Detailed information regarding each participant’s nutritional status encompassed the assessment of the following anthropometric measurements: body weight; standing height; mid-upper arm, waist, and calf circumferences; triceps skinfold thickness; and the functional status indicators of hand grip strength and walking speed [30,31].

Anthropometric measurements were collected following standard procedures [32]. Standing height was obtained with a calibrated stadiometer (Seca 213) with 0.1-centimeter resolution. For participants with visible kyphosis, or when it was impossible to measure standing height due to the participant’s paralysis, mobility, or balance limitations, height was obtained indirectly from nondominant hand length (in centimeters) [33], measured with a calibrated paquimeter (Fervi Equipment) with 0.1-centimeter resolution. Body weight (in kilograms) was measured with a calibrated portable electronic scale (Seca 803) with 0.1-kilogram resolution, with the participants wearing light clothing. When it was not possible to weigh a patient (for the same reasons described for standing height measurement) body weight was estimated from mid-upper arm and calf circumferences [34]. Mid-upper arm, waist, and calf circumferences were measured with a metal tape measure (Lufkin) with 0.1-centimeter resolution. Triceps skinfold thickness was obtained using a Holtain Tanner/Whitehouse skinfold caliper with 0.2-millimeter resolution.

Nondominant hand grip strength was measured with a calibrated Jamar Hand Dynamometer (Sammons Preston), as recommended by the American Society of Hand Therapists [35]. Each participant performed three measurements with a one-minute pause between measurements [36]. When the individual was unable to perform the measurement with the nondominant hand, the dominant hand was used.

The MNA-SF consists of six questions targeting food intake, weight loss, physical and mental status, and anthropometry through body mass index (BMI) assessment. BMI was calculated using the standard formula (weight in kilograms/height^2^ in meters). A participant scoring ≦7 out of 14 points was classified as undernourished, one that scored between 8 and 11 was at risk of undernutrition, and one scoring between 12 and 14 points was considered well-nourished [30].

Frailty, according to the frailty phenotype described by Fried et al [37], encompasses the assessment of five criteria: unintentional weight loss in the previous year, weakness evaluated as low hand grip strength (adjusted for gender and BMI), poor endurance and energy evaluated as exhaustion, slowness (gait speed measurement adjusted for gender and standing height), and low activity (kilocalories expended per week, adjusted for gender). If one or two of these criteria were present, the individual was characterized as prefrail. Frailty was defined as the presence of three or more criteria [37].

According to the European Working Group on Sarcopenia in Older People [38], sarcopenia was defined as the combined presence of low muscle mass and low muscle strength, or diminished physical performance. Muscle mass was assessed based on the two compartment model (body muscle mass = body weight - body fat mass). Body density was estimated based on triceps skinfold thickness [39] and body density was converted to fat mass through the Brozek equation [40]. Muscle strength was evaluated by hand grip strength (adjusted for gender and BMI) and physical performance by gait speed. Presarcopenia occurs when only muscle mass is diminished. Sarcopenia is characterized by low muscle mass plus one of the other two criteria. All three criteria are present in an individual with severe sarcopenia.

Information on cohabitation, skin phenotype (as measured by the Fitzpatrick classification [41]), and household income were also collected.

### Laboratory Procedures and Biological Samples

A sample of blood and the volume of urine in a 24-hour period were collected for each participant. The study interviewers gave the participants oral and written instructions detailing the collection and storage procedures for the volume of 24-hour urine. Participants were instructed to refrain from collecting the first urine of the day, but to record the time of the first urine, and collect all excreted urine during the day and evening. The following day, participants collected the morning urine until the time they recorded the first urine the day before. A 24-hour urine container was also provided, and participants were instructed to keep the container in the refrigerator until it was delivered for analysis. A certified laboratory (Labco Portugal) was responsible for blood and urine sample collection and analyses.

Vitamin D status was evaluated by dosing the plasmatic levels of 25-hydroxycholecalciferol or calcidiol through the electrochemiluminescence immunoassay using Roche Cobas Vitamin D total assay reagent (Roche Diagnostics GmbH, Mannheim, Germany). Blood samples for these analyses were collected by qualified nurses within four days of the application of the questionnaire, and preferentially after a 12-hour fasting period.

The following urinary markers were quantified: urine volume (milliliters), urinary creatinine (milligrams/day), urine osmolality (milliosmoles/kg), and urine density for 24 hours. Urinary creatinine was measured by the Jaffe method.

Hydration status was evaluated by free water reserve (milliliters/24 hours [42-47]) calculated by subtracting 24-hour urine volume from obligatory urine volume (solutes in urine 24 hours [milliosmoles/day] / 830 - 3.4 x [age - 20]), allowing for the classification of the 24-hour hydration status (euhydrated vs hypohydrated subjects, or at risk of hypohydration [43,48]). Urine samples were also analyzed for urinary sodium (milliequivalents/day); however, for comparative purposes, these values were converted to milligrams/day by using the molecular weight of sodium (23 milligrams sodium = 1 millimole sodium or 1 milliequivalents sodium).

## Results

Data collection (n=1500) took place between December, 2015 and June, 2016 and results are being analyzed. Final results will be disseminated by scientific journals and via the media throughout Portugal.

## Discussion

Nutrition UP 65 will provide an innovative and important contribution to overcome the lack of data regarding nutritional conditions in Portugal. These data will also generate information to define public health interventions and guidelines tailored to Portugal’s health realities.

This project also expects to bridge the gap in knowledge regarding the country’s regional differences with respect to the prevalence of inadequate nutritional status, particularly in rural areas, by the gathering of nationwide nutritional information (including rural areas and the interior region). Nutrition UP 65 includes a sample of older adults that are widely distributed in different geographical regions, thus allowing for a *picture* of the country’s situation. This study will first describe the nutritional status of older populations according to regional area, and using the same methodology, provide a better understanding on nutritional risk contrasts. This baseline nutritional status description will support the development of evidence-based public action that considers regional discrepancies and contrasts. This study will make it possible to define the main regional priorities for nutritional intervention at the level of primary health care, hospitals, and community.

The data from this project will reveal a nationwide description of the burden of major nutritional health problems affecting older Portuguese adults, identify vulnerable target groups for public health interventions, and allow for the implementation of an evidence-based nutritional surveillance system. Nutrition UP 65 will guide the design and implementation of preventive public health strategies at all levels of dependence, with unequivocal health gains for this population group. These strategies have been proven to be economically effective, and increase the awareness of health professionals with regards to nutrition-related issues.

Furthermore, information regarding the main nutritional problems that affect older populations will empower older adults with knowledge to recognize nutritional imbalances, and to have better nutrition, which should help to prevent major nutritional problems and nutrition-related disabilities.

## Appendix

Multimedia Appendix 1 Sample size and constitution.

## Abbreviations

BMI: body mass index
MNA-SF: Mini-Nutritional Assessment - Short Form
